# Endodontic Emergencies in Mumbai City during COVID-19 Lockdown and Different Phases of Unlock

**DOI:** 10.3390/ijerph18147314

**Published:** 2021-07-08

**Authors:** Omkar Shinde, Ajinkya M. Pawar, Kulvinder Singh Banga, Jatin Atram, Dian Agustin Wahjuningrun

**Affiliations:** 1 Department of Conservative Dentistry and Endodontics, Nair Hospital Dental College, Mumbai 400034, Maharashtra, India; omkarshinde0206@gmail.com (O.S.); ajinkya@drpawars.com (A.M.P.); ksbanga@gmail.com (K.S.B.); jatin.atram2008@gmail.com (J.A.); 2 Department of Conservative Dentistry, Faculty of Dental Medicine, Universitas Airlingga, Surabaya City 60132, Indonesia

**Keywords:** COVID-19, endodontic emergency, epidemiologic investigation, verbal numerical rating scale, vital pulp therapy, lockdown, Mumbai metropolitan region

## Abstract

The present descriptive research focused on determining the traits of patients who required endodontic treatment during the COVID-19 lockdown and various stages of unlocking in Mumbai metropolitan region. The descriptive analysis was carried out by examining the patients who were registered at Nair Hospital Dental College, Mumbai during four phases of lockdown (25 March to 31 May 2020) and eight phases of unlock (1 June 2020 to 31 January 2021). The evaluation was performed by evaluating the number of days and the overall number of patients reported for endodontic treatment for the first and subsequent visits. Each patient underwent intensive triage, which included taking their body temperature, oxygen saturation level, and travel history. The sex, age, and endodontic diagnosis of the offending tooth were also reported. The pain parameters were analyzed using a verbal numerical rating score (VNRS). During the lockdown phases, 297 patients seeking endodontic treatment visited the hospital over a total of 26 working days, and during the unlock phases, 16,195 patients visited the hospital over 189 working days. The average age of the patients that visited were 31–40 years of age (57.23%). The mean VNRS score was 5.85 ± 1.62. For both the lockdown and unlock periods, a significantly higher percentage of males visited dental school (*p* < 0.001). When compared to other stages, the number of patients attending during lockdown phase 4 (55.56%) and unlock phase 8 (32.35%) was the highest. The majority of endodontic emergency diagnoses were reversible pulpitis (36.5%) and symptomatic irreversible pulpitis (33.14%), all of which had higher mean VNRS (*p* < 0.05). Of the 49 patients that reported with COVID-19 symptoms, 11 tested positive. During the lockdown and unlock periods, 12 of the 41 treating workers tested positive. Personal protective equipment and patient screening are critical in shielding clinicians during the COVID-19 pandemic.

## 1. Introduction

The COVID-19, which is caused by the Severe Acute Respiratory Syndrome Coronavirus 2 (SARS-CoV-2) exhibits symptoms such as fever, dry cough, nausea, difficulty breathing, and shortness of breath [1]. On 30 December 2019, several COVID-19 cases were registered in China’s Wuhan Province [2]. On 11 March 2020, the World Health Organization (WHO) declared it a global pandemic [3]. Since then, every part of human life has been influenced. COVID-19 spreads by sneezing and coughing, nasal droplets and polluted surfaces, and the mucous membranes of the lips, skin, and nose [4].

On 30 January 2020, India reported the first case of COVID-19 in the city of Thrissur, Kerala. By February 3, two more cases of students returning to India from China’s Wuhan Province had been identified. There was no evidence of a significant increase in recent COVID-19 cases over the next few days. At the beginning of March 2020, the number of new cases of COVID-19 in India increased significantly. On 9 March 2020, the city of Pune identified the first confirmed COVID-19 case in Maharashtra. Following that, new cases had been registered in major Maharashtra cities such as Mumbai, Nagpur, and Ahmednagar [5].

By the mid of March 2020, owing to the number of active cases of COVID-19 in India, the Prime Minister of India announced a nationwide lockdown for 21 days on 24 March 2020, at 8 p.m., to control the further spread of the virus [6]. The national lockdown put restrictions on the movement of 1.3 billion people. Only medical emergencies were initially permitted. Even after the announcement of the national lockdown, new cases were reported by the city of Mumbai at an alarming rate, more than 40 cases per day, up to a maximum of 3659 cases on a single day (21 August 2020), compared to other regions of Maharashtra [7].

Endodontic infections can be extremely painful, and endodontic emergencies are a common form of dental emergency. In patients who needed endodontic care, there was no assurance that the same would be handled during the lockdown. The Nair Hospital Dental College was one of the few hospitals that provided emergency dental care. During the period of lockdown, emergency treatment was provided in the emergency ward located on the first floor of the dental hospital. Eventually, at the end of the second phase of unlock i.e., on 29 July, the hospital turned into a full-fledged OPD and offered treatment in all the departments.

The aim of the current descriptive analysis was to report the characteristics of patients visiting the department of Conservative Dentistry and Endodontics at Nair Hospital Dental College, Mumbai, with a primary complaint suggestive of endodontic origin. The objective of the analysis was to figure out the pattern of such patients during the four phases of lockdown (25 March 2020 to 31 May 2020) and eight phases of unlock (1 June 2020 till 31 January 2021).

## 2. Materials and Methods

### 2.1. Study Setting

Nair Hospital Dental College, Mumbai is an integrated healthcare organization. Its comprehensive health records store linked information on all aspects of dental care for each patient across all care settings (for example, outpatient, inpatient, and the emergency department). Each patient was assigned a unique medical record number that allowed for the linkage of data across all dental care aspects. Nair Hospital Dental College had a diverse patient population from Mumbai Metropolitan Region (MMR) and other locations.

### 2.2. Study Design and Patients

We performed a retrospective cohort study on people who consecutively visited the emergency department (ED) and were referred to the Department of Conservative Dentistry and Endodontics of Nair Hospital Dental College 15 April–31 May 2020. The patients were divided into lockdown/ED (25 March 2020–31 May 2020) and unlock/Out-Patient Department (OPD) of Conservative and Endodontics (1 June 2020–31 January 2021). We followed up with patients until their second visit.

### 2.3. Data Sources

**Figure 1:**
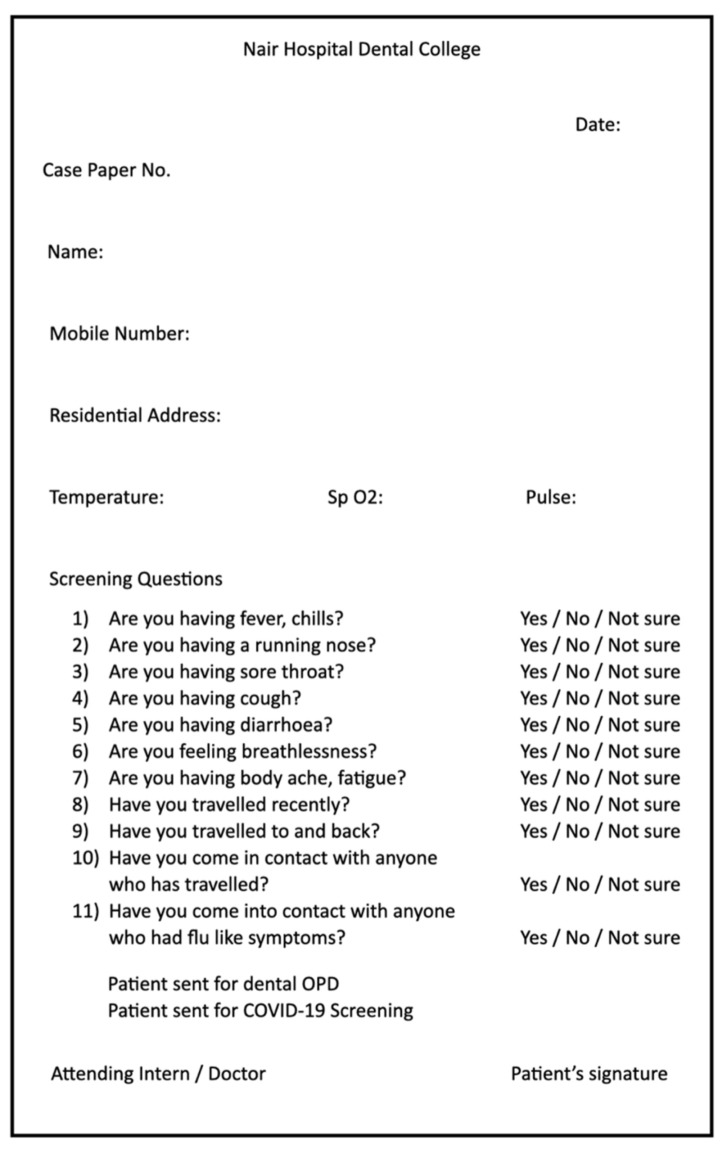
Patient triage form that was recorded for each patient.

We obtained information from the Nair Hospital Dental College, Mumbai, database. The database contains data for inpatient and outpatient visits at Nair Hospital Dental College, Mumbai, including demographic characteristics, diagnoses, procedures, medications, and laboratory tests. After the inception of the COVID-19 pandemic, each patient was subjected to a thorough examination of their body temperature, pulse rate, oxygen saturation, and travel history (Figure 1). We excluded patients who showed COVID-19 symptoms. The diagnoses were rendered using the American Board of Endodontics and the American Association of Endodontics consensus guidelines [8]. Diagnoses were extracted from both the inpatient and outpatient records.

### 2.4. Ethical Approval

Ethical approval for the execution of the descriptive analysis was obtained based on the Declaration of Helsinki from the Nair Hospital Dental College Institutional Ethics Committee (EC-ND-156/2020, dated: 19 September 2020). Permission for the project was also obtained from the principal and head of the department. 

### 2.5. Statistical Analysis

The data was entered and analyzed using the Statistical Package for Social Sciences (SPSS) for Windows 26.0. (SPSS, Inc. Chicago, IL, USA) Descriptive analysis was done to compare patients between first and second visits and patients during the lockdown and unlock phases.

## 3. Results

The lockdown and unlock duration were divided into four and eight phases, respectively (Table 1). During lockdown, Nair Hospital Dental College had 26 working days, and a total of 297 patients visited the hospital. No patients visited during phase 1 lockdown (25 March to 14 April 2020). However, during the different phases of unlock over 189 working days, 16,195 patients visited the hospital. The patient flow drastically increased during unlock phases. The percentage of patients visiting during lockdown phase 4 (165 of 297; 55.56%) and unlock phase 8 (5238 of 16,195; 32.35%), was highest compared to other phases (Table 1).

The frequency of males visiting the dental hospital was much higher than females during the lockdown (256 out of 297 i.e., 86.19%) and unlock phases (10,201 out of 16,195 i.e., 63%). Only 1 patient visited the dental hospital during lockdown aged above 50 years, which can be attributed to the high risk of COVID-19 infection. The highest number of patients who visited during lockdown 4 (243 out of 297) and unlock 8 (9196 out of 16,195) phases belonged to the 31–40-years-old group (Table 2). 

The mean VNRS for different sex, age and endodontic emergencies is presented in Table 3. Patients in the age group 31–40-years-old and with an endodontic emergency related to reversible pulpitis and symptomatic irreversible pulpitis exhibited significantly higher VNRS (*p* < 0.05).

A total of 194 and 4471 patients were referred for endodontic care during the lockdown and unlock phases, respectively, for their first visit. Also, 103 and 11,724 patients during the lockdown and unlock phases, respectively, were attending the hospital for a follow-up visit and required continuation of their endodontic treatment. Reversible pulpitis and symptomatic irreversible pulpitis were the most common chief complaints during the lockdown and unlock phases of the first and follow-up visit. The highest number of patients were referred during the lockdown phase 4 (55.17%) and unlock phase 8 (25.16%) for their first visit. Furthermore, 56.32% of the patients were referred during lockdown phase 4, and 35.13% of the patients were referred during unlock phase 8 for the follow-up or second visit (Table 4 and Table 5). The patient flow was higher for both lockdown and unlock phases from the Mumbai metropolitan region (MMR) compared to non-MMR region (Table 6).

The lockdown resulted in a low number of trauma (complicated crown fracture and avulsion) cases, which was 12, compared to the number of cases reported during the unlock phases, which was about 205. A total of 1516 patients who had a chief complaint of acute apical abscess were reported during lockdown and unlock phases. Out of these, 424 patients had a temperature of more than 37.2 °C. All of them were found to be safe from COVID-19 infection after proper questionnaire evaluation.

Of the 16,492 patients treated, 49 presented with COVID-19 symptoms during the triage process and were referred for RT-PCR testing, and 11 individuals tested positive. Additionally, of the 41 healthcare workers (HCW) that were posted while rendering treatments during lockdown and unlock, 12 tested positive.

## 4. Discussion

SARS-CoV-2, the virus that causes COVID-19, has an average incubation period of 2 to 14 days. Patients that have no symptoms or are in the incubation process are thought to be able to infect others [9]. Despite the fact that COVID-19 active cases were on the rise in Maharashtra and Mumbai at the beginning of March 2020, all dental schools remained open and operational as normal. In the second half of March 2020, India’s Prime Minister announced a national shutdown, prompting all to close, including dental colleges, for 21 days. The Municipal Corporation of Greater Mumbai (MCGM)-founded Nair Hospital Dental College (NHDC) in Mumbai was one of the country’s only dental schools that offered emergency dental care during the lockdown. The estimated number of patients declined significantly as a result of the limitations imposed during the lockdown. Furthermore, since other dental schools were closed during the lockdown, the number of first-time patients visiting NHDC rose (194 first visits and 103 second visits out of 297 patients).

During the lockdown periods, the total number of patients visiting the emergency ward for endodontic care was 297 over 26 working days, while the total number of patients visiting the Department of Endodontics was 16,195 over 189 working days. A substantial number of patients visited the dental school during the lockdown, and this number gradually increased as the unlocking process progressed. The importance of patient screening (Figure 1) during these processes was emphasized, and it is still necessary today. This was done by a COVID-19 disease-specific questionnaire, which possessed all the basic information on the patients’ demographics (if the patient was from a hotspot or no), history of travel, contact with any COVID-19-positive patient, and any symptoms of COVID-19, like fever, chills, cough, and cold.

None of the patients were reported with any symptoms of COVID-19 as evaluated by the specific questionnaire during the lockdown. However, 49 patients during the unlock phase were reported to have symptoms and were referred to Topiwala National Medical College, Mumbai (TNMC) for reverse transcription polymerase chain reaction (RT-PCR) test. Out of these 49 patients, 38 returned to the department and presented a negative report of COVID-19 infection. The other 11 patients failed to report to the department. On telephonic communication, they were reported to be positive with COVID-19 infection and were admitted to COVID care centers (CCC) close to their residence. These patients resided in various hotspots (enlisted by MCGM) of the city namely, three from Dharavi, four from Worli, one from Borivali, and one from Mulund. Only two patients that were tested positive were not from the hotspots or containment zones. As a result, the disease-specific questionnaire proved to be a valuable tool for patient screening and preventing the spread of COVID-19 infection among healthcare staff (HCW) serving NHDC.

No patients had confirmed or suspected asymptomatic COVID-19 infection or were experiencing COVID-19 symptoms. A greater number of male patients were reported during the lockdown (256 out of 297 patients) and until phase 5 of unlock (1121 out of 1598 patients). It eventually changed to an almost similar number of males and females in the Phase 7 of unlock. This could be due to the relaxation and permission to use the public transport at a speculated given time. The visitors to the dental school ranged in age from 13 to >60 years. Patients in the oldest age groups (51 to 60 years = 904 out of 16,492 patients and >60 years = 552 out of 16,492 patients) had the lowest rate of coming in for treatment during the lockdown and unlock phases. This may be due to the younger family members and their own knowledge of the existence of comorbidities and the associated weaker COVID-19 prognosis [10,11].

Fever was recorded in a total of 28% patients (424 out of 1516 patients of lockdown and unlock period) with acute apical abscess who had a body temperature of more than 37.2 °C. They were found to be safe of COVID-19 infection after the questionnaire evaluation, and thus, their fever was identified as one of the systemic manifestations of acute apical abscess [12]. A highly recommended Q COVID-19 Ag test was performed on these patients [13], which showed up negative. As a consequence, a fever alone should not be the only sign or symptom of COVID-19 to be considered. COVID-19 is currently diagnosed using a combination of epidemiologic data, clinical symptoms, chest computed tomographic imaging results, and laboratory tests, including RT-PCR respiratory tract specimens.

The following measures were taken in the lockdown phase, when cases of COVID-19 were rising at alarming numbers. The most common endodontic emergency reported during the lockdown and unlock was reversible pulpitis, followed by symptomatic irreversible pulpitis, symptomatic apical periodontitis, acute apical abscess, chronic apical abscess, complicated crown fracture, and avulsion. The rendering of treatment to these endodontic emergencies is highly risky due to the fact that these treatments are high-aerosol generating. Reducing treatment time and exposure control are two ways the risk of infection for endodontic treatment could be reduced. In cases with vital pulps, pulpotomy was considered a treatment option, as it is shown to reduce pain considerably, providing comfort to the patient [14]. Another option that was considered was partial pulpotomy using mineral trioxide aggregate (MTA) for cases with irreversible pulpitis [15]. This option has shown promising results in permanent teeth with multiple years of follow-up. For patients requiring root-canal treatments, the use of single file systems was preferred as it substantially reduced the working time and prevented the risk from resterilization. As the treatment was provided by experienced endodontists (minimum 6 years), exposing an IOPA radiograph was avoided. The IOPA radiograph (placing of sensor/films) can stimulate saliva secretion, coughing, nausea, and vomiting and causes exposure to patients’ oral cavity [16]. The favorable drugs of choice that were prescribed for patients with severe dental pain for treating acute pulpitis were acetaminophen (1000 mg every 6–8 h), ketorolac tromethamine (10 mg every 6 h), or piroxicam (20 mg every 12 h) [17]. Additionally, in patients experiencing pain suffering from symptomatic irreversible pulpitis, oral dexamethasone (4 mg) was prescribed to reduce the pain considerably [18]. Due to the conflicting research on ibuprofen usage during COVID-19, it was not prescribed to the patients [19]. As the treatment procedures generated droplets and aerosols, the mandatory use of a rubber dam was practiced, as it has been shown to reduce these issues by about 70% around a 3 feet diameter operating area [20].

Treatment was provided taking into consideration the guidelines of Center for Disease Prevention and Control (CDC) by keeping no patients in the waiting area of the department. Socially distant office chairs were placed in the parking lot, and the patient was allowed to come to the department floor only when his appointment time was reached. Care was also taken to avoid any overlap of appointments for each operator in the department. The treatment was provided to the patients on alternate dental chairs, ensuring a distance of more than the recommended 2 m (6 feet) in each direction [21]. Additionally, as recommended by WHO, 5% sodium hypochlorite, in a 1:100 dilution, was applied on surfaces after the dental procedure for 10 min [22].

There was also a significant reduction in physical accidents. The reduction of outdoor activities during the lockdown may have consequently resulted in a decrease in trauma occurrences. During the four phases of lockdown, only 12 patients (first visit: five complicated crown fractures and one avulsion; second visit: four complicated crown fractures and two avulsions) presented traumatic injuries. During the different phases of unlock, the traumatic injury cases increased to 45 complicated crown fractures and 28 avulsions (first visit), and 83 complicated crown fractures and 49 avulsions (second visit). The reduced number of traumatic injuries during COVID-19 could be due to the restrictions for travel and the limited availability of public transport to only healthcare and essential workers.

During the unlock phases, the number of patients reporting to the dental hospital increased as the cases of COVID-19 fell and there was a relaxation of the travel requirements. Treatments to these patients were provided with all the measures, from the triage screening to the wearing of PPE and restricting the time of exposure of the endodontists, post-graduate students, nursing staff, and auxiliary staff to the patients’ oral cavity.

A total of 41 HCWs, which included teaching staff, post-graduate students, interns, under-graduate students, nursing staff, servants, and clerks in the Department of Conservative Dentistry and Endodontics, were taking appropriate safety precautions when handling patients directly or indirectly. At all times, they wore N95 masks, gloves, caps, shoe covers, face shields, and personal protective gowns. Direct transmission (via cough, sneeze, or droplet inhalation), touch transmission (via the oronasalocular route), and aerosol transmission are the three most common modes of transmission for COVID-19 [23]. Furthermore, due to the high risk of aerosol production during dental treatment, the operatories were properly ventilated and fitted with HEPA filter air purifiers, which has been shown to minimize dental healthcare worker exposure to aerosols by 80–95% [17,24]. Since the lockdown, 12 HCWs have tested positive for COVID-19 infection, including three teaching faculty, three post-graduate students, one intern, three undergraduate students, and two servants. The safety precautions and screening measures introduced since the announcement of lockdown (March 25, 2020) have proven to be successful in protecting HCWs, with just a few cases of positive COVID-19 infections while providing care.

### 4.1. Strength

For the first time in scientific literature, this study has presented such an important issue. Endodontic situations account for a considerably large proportion of dental emergencies, even during the COVID-19 pandemic. During the COVID-19 outbreak, the mandatory use of rubber dams, personal protective equipment, and patient screening all play an essential role in keeping healthcare workers safe from contracting the infection.

### 4.2. Limitations

The current analysis was limited to a single department of an institute, which consisted of only endodontic care. During the COVID-19 outbreak, patients’ psychological well-being should also be assessed.

### 4.3. Challenges

The current study had various challenges: communicating to the patients who reported with COVID-19 symptoms, reduced contact time with patients, and most importantly, fear of contracting the infection.

## 5. Conclusions

In the current descriptive analysis, a total of 16,492 (297—lockdown and 16,195—unlock) patients reported to the dental hospital for various endodontic treatments. The average age group of the patients was between 31 and 40 years. The majority of endodontic emergency diagnoses for the patients reported were reversible pulpitis and symptomatic irreversible pulpitis. While rendering treatment to a total of 16,492 patients during, 49 patients reported with COVID-19 symptoms at the primary screening triage, of which 11 tested positive. A total of 12 of the 41 dental, nursing, and auxiliary staff tested positive during the evaluated duration. The low number of infected HCWs was due to the treatments that were performed sticking to the ideal guidelines of practicing dentistry during COVID-19. The recent COVID-19 pandemic challenges the existing clinical procedures. Our prime focus as dental healthcare professionals is to assist our patients in their time of need. Reducing the treatment time and exposure control are two ways to significantly reduce the risk of COVID-19 spreading during endodontic treatment.

## Figures and Tables

**Table 1:** The number of working days, number, and percentages (%) of the patients visited the dental school for their first and follow-up visit during the different lockdown and unlock phases.

**Lockdown Phases**	**No. of Working Days (26 Days)**	**First Visit** **(% of 194)**	**Follow-Up Visit** **(% of 103)**	**Total** **(% of 297)**
**Phase 1** 25 March to 14 April 2020	0	0	0	0
**Phase 2** 15 April to 3 May 2020	6	29 (14.94%)	13(12.62%)	42 (14.14%)
**Phase 3** 4 May to 17 May 2020	9	58 (29.89%)	32(31.06%)	90 (30.30%)
**Phase 4** 18 to 31 May 2020	11	107 (55.17%)	58(56.32%)	165 (55.56%)
**Unlock Phases**	**No. of Working Days (189 Days)**	**First Visit** **(% of 4471)**	**Follow-Up Visit** **(% of 11,724)**	**Total** **(% of 16,195)**
**Phase 1** 1 June to 30 June 2020	12	125 (2.79%)	103 (0.87%)	228 (1.40%)
**Phase 2** 1 July to 31 July 2020	25	244 (5.45%)	481(4.10%)	725 (4.47%)
**Phase 3** 1 August to 31 August 2020	27	507 (11.33%)	708 (6.03%)	1215 (7.50%)
**Phase 4** 1 September to 30 September 2020	23	673 (15.05%)	686 (5.85%)	1359 (8.39)
**Phase 5** 1 October to 30 October 2020	26	595 (13.30%)	1003 (8.55%)	1598 (9.86%)
**Phase 6** 1 November till 30 November 2020	25	503 (11.25%)	1897 (16.18%)	2400 (14.81%)
**Phase 7** 1 December till 31 December 2020	24	701 (15.67%)	2731 (23.29%)	3432 (21.19%)
**Phase 8** 1 January till 31 January 2021	27	1123 (25.16%)	4115 (35.13%)	5238 (32.35%)

**Table 2:** Demographic data with respect to the sex and age of patients that visited the dental school during the different lockdown and unlock phases.

**Lockdown**	**Sex**		**Age**						**Total**
	**Male**	**Female**	**13–20**	**21–30**	**31–40**	**41–50**	**51–60**	**>60**	
**Phase 1**	0	0	0	0	0	0	0	0	0
**Phase 2**	35	7	2	2	35	2	1	0	42
**Phase 3**	76	14	7	9	68	6	0	0	90
**Phase 4**	145	20	4	17	140	4	0	0	165
**Total**	256	41	13	28	243	12	1	0	297
**Unlock**	**Sex**		**Age**						**Total**
	**Male**	**Female**	**13–20**	**21–30**	**31–40**	**41–50**	**51–60**	**>60**	
**Phase 1**	201	27	9	16	193	7	2	1	228
**Phase 2**	603	122	25	65	570	29	21	15	725
**Phase 3**	980	235	83	165	798	71	59	39	1215
**Phase 4**	1031	328	92	183	853	95	85	51	1359
**Phase 5**	1121	477	113	265	912	131	105	72	1598
**Phase 6**	1695	705	228	375	1319	221	159	98	2400
**Phase 7**	1867	1565	353	527	1825	415	187	125	3432
**Phase 8**	2703	2535	525	753	2726	798	285	151	5238
**Total**	10,201	5994	1428	2349	9196	1767	903	552	16,195

**Table 3:** The total number of patients with different sex, age, endodontic emergencies, and the mean VNRS for those who visited the dental school during both of the phases.

Variables	*p* Value	Number (%)	Mean VNRS Score	*p* Value of VNRS
**Sex**				
Male #	<0.05	10,457 (63.40%)	6.56 ± 1.47	>0.05
Female		6035 (36.60%)	6.27 ± 1.53	
**Age**				
13–20	<0.05	1441 (8.73%)	5.01± 1.58	<0.05
21–30		2377 (14.41)	5.23 ± 1.49	
31–40		9439 (57.23%)	5.85 ± 1.62 *	
41–50		1779 (10.79%)	5.16 ± 1.34	
51–60		904 (5.48%)	4.79 ± 1.43	
>61		552 (3.36%)	4.54 ± 1.33	
**Diagnosis**				
Reversible Pulpitis	<0.05	6021 (36.29%)	5.45 ± 1.35 *	<0.05
Symptomatic Irreversible Pulpitis		5467 (32.94%)	5.37 ± 1.46 *	
Symptomatic Apical Periodontitis		1921 (11.56%)	5.11 ± 1.51	
Chronic Apical Abscess		1451 (8.74%)	5.02 ± 1.64	
Acute Apical Abscess		1516 (9.14%)	5.04 ± 1.53	
Complicated Crown Fracture		137 (0.83%)	3.85 ± 0.56	
Dislocation of Tooth		80 (0.50%)	3.01 ± 0.02	

The values marked with (**#**) exhibited significantly higher number of patients according to sex and those marked with (*) exhibited significantly higher VNRS (*p* < 0.05) amongst the different age groups and types of endodontic emergency.

**Table 4:** Number of patients that visited the dental school for their first visit for different endodontic emergencies during lockdown and unlock phases.

**Lockdown**	**Reversible** **Pulpitis**	**Symptomatic** **Irreversible** **Pulpitis**	**Symptomatic Apical** **Periodontitis**	**Chronic Apical Abscess**	**Acute Apical Abscess**	**Complicated Crown Fracture**	**Avulsion**	**Total** **(% of 194)**
**Phase 1**	0	0	0	0	0	0	0	0
**Phase 2**	18	4	3	2	1	1	0	29 (14.94%)
**Phase 3**	33	8	7	5	4	1	0	58 (29.89%)
**Phase 4**	57	15	11	9	11	3	1	107 (55.17%)
**Unlock**	**Reversible Pulpitis**	**Symptomatic Irreversible Pulpitis**	**Symptomatic Apical** **Periodontitis**	**Chronic Apical Abscess**	**Acute Apical Abscess**	**Complicated Crown Fracture**	**Avulsion**	**Total** **(% of 4471)**
**Phase 1**	72	22	4	13	7	6	1	125 (2.79%)
**Phase 2**	100	66	23	26	24	4	1	244 (5.45%)
**Phase 3**	190	132	55	59	64	5	2	507(11.33%)
**Phase 4**	254	179	99	63	71	5	2	673(15.05%)
**Phase 5**	225	203	45	88	25	5	4	595(13.30%)
**Phase 6**	169	166	54	52	52	6	4	503(11.25%)
**Phase 7**	324	212	81	86	84	7	7	701(15.67%)
**Phase 8**	384	307	145	92	181	7	7	1123(25.16%)

**Table 5:** Number of patients that visited the dental school for their second/follow-up visit for different endodontic emergencies during the lockdown and unlock phases.

**Lockdown**	**Reversible** **Pulpitis**	**Symptomatic** **Irreversible** **Pulpitis**	**Symptomatic Apical** **Periodontitis**	**Chronic Apical Abscess**	**Acute Apical Abscess**	**Complicated Crown Fracture**	**Avulsion**	**Total** **(% of 103)**
**Phase 1**	0	0	0	0	0	0	0	0
**Phase 2**	6	1	1	2	2	1	0	13 (12.62%)
**Phase 3**	11	8	4	5	3	1	1	32 (31.06%)
**Phase 4**	15	16	9	8	7	2	1	58 (56.32%)
**Unlock**	**Reversible Pulpitis**	**Symptomatic Irreversible Pulpitis**	**Symptomatic Apical** **Periodontitis**	**Chronic Apical Abscess**	**Acute Apical Abscess**	**Complicated Crown Fracture**	**Avulsion**	**Total** **(% of 11,724)**
**Phase 1**	30	31	17	12	10	2	1	103 (0.87%)
**Phase 2**	143	141	65	51	71	5	5	481 (4.10%)
**Phase 3**	249	245	31	51	124	5	3	708 (6.03%)
**Phase 4**	225	224	67	69	89	7	5	686 (5.85%)
**Phase 5**	359	368	61	81	117	12	5	1003 (8.55%)
**Phase 6**	696	655	227	145	154	11	9	1897(16.18%)
**Phase 7**	1004	1052	321	196	129	19	10	2731(23.29%)
**Phase 8**	1457	1412	591	336	286	22	11	4115(35.13%)

**Table 6:** Number of patients that visited the dental school from the Mumbai metropolitan region and non-Mumbai metropolitan region during lockdown and unlock phases.

**Lockdown**	**Mumbai** **Metropolitan** **Region**	**Non-Mumbai** **Metropolitan** **Region**	**Total** **(% of 297)**
**Phase 1**	0	0	0
**Phase 2**	37	5	42 (14.14%)
**Phase 3**	82	8	90 (30.30%)
**Phase 4**	145	20	165 (55.56%)
**Unlock**	**Mumbai** **Metropolitan** **Region**	**Non-Mumbai** **Metropolitan** **Region**	**Total** **(% of 16,195)**
**Phase 1**	197	31	228 (1.40%)
**Phase 2**	556	169	725 (4.47%)
**Phase 3**	910	305	1215 (7.50%)
**Phase 4**	1107	525	1359 (8.39%)
**Phase 5**	960	638	1598 (9.86%)
**Phase 6**	1302	1098	2400 (14.81%)
**Phase 7**	2011	1421	3432 (21.19%)
**Phase 8**	3035	2203	5238 (32.38%)

## Data Availability

The data is the property of the Department of Conservative Dentistry and Endodontics, Nair Hospital Dental College, under K.S.B. and he should be contacted if the data needs to be shared.

## References

[1] Zhu N., Zhang D., Wang W., Li X., Yang B., Song J., Zhao X., Huang B., Shi W., Lu R. (2020). A Novel Coronavirus from Patients with Pneumonia in China, 2019. N. Engl. J. Med..

[2] Lauer S.A., Grantz K.H., Bi Q., Jones F.K., Zheng Q., Meredith H.R., Azman A.S., Reich N.G., Lessler J. (2020). The Incubation Period of Coronavirus Disease 2019 (COVID-19) From Publicly Reported Confirmed Cases: Estimation and Application. Ann. Intern. Med..

[3] Gallegos A. WHO Declares Public Health Emergency for Novel Coronavirus. Medscape Medical News 2020. https://www.medscape.com/viewarticle/924596.

[4] Dhand R., Li J. (2020). Coughs and Sneezes: Their Role in Transmission of Respiratory Viral Infections, Including SARS-CoV-2. Am. J. Respir. Crit. Care Med..

[5] Wikipedia Contributors COVID-19 Pandemic in Maharashtra. Wikipedia. https://en.wikipedia.org/wiki/COVID-19_pandemic_in_Maharashtra.

[6] Wikipedia Contributors COVID-19 Lockdown in India. Wikipedia. https://en.wikipedia.org/wiki/COVID-19_lockdown_in_India.

[7] Malishery S., Pawar A.M., Atram J. (2020). Efforts to Curb the COVID -19 Pandemic: An Example from Mumbai and Maharashtra, India. IJARESM.

[8] Glickman G.N. (2009). AAE Consensus Conference on Diagnostic Terminology: Background and perspectives. J. Endod..

[9] Chen N., Zhou M., Dong X., Qu J., Gong F., Han Y., Qiu Y., Wang J., Liu Y., Wei Y. (2020). Epidemiological and clinical characteristics of 99 cases of 2019 novel coronavirus pneumonia in Wuhan, China: A descriptive study. Lancet.

[10] Meng L., Hua F., Bian Z. (2020). Coronavirus Disease 2019 (COVID-19): Emerging and Future Challenges for Dental and Oral Medicine. J. Dent. Res..

[11] Samaranayake L.P., Peiris M. (2004). Severe acute respiratory syndrome and dentistry: A retrospective view. J. Am. Dent. Assoc..

[12] Siqueira J.F., Rôças I.N. (2013). Microbiology and treatment of acute apical abscesses. Clin. Microbiol. Rev..

[13] Ristić M., Nikolić N., Čabarkapa V., Turkulov V., Petrović V. (2021). Validation of the STANDARD Q COVID-19 antigen test in Vojvodina, Serbia. PLoS ONE.

[14] Hasselgren G., Reit C. (1989). Emergency pulpotomy: Pain relieving effect with and without the use of sedative dressings. J. Endod..

[15] Taha N.A., Khazali M.A. (2017). Partial Pulpotomy in Mature Permanent Teeth with Clinical Signs Indicative of Irreversible Pulpitis: A Randomized Clinical Trial. J. Endod..

[16] Vandenberghe B., Jacobs R., Bosmans H. (2010). Modern dental imaging: A review of the current technology and clinical applications in dental practice. Eur. Radiol..

[17] Krithikadatta J., Nawal R.R., Amalavathy K., McLean W., Gopikrishna V. (2020). Endodontic and dental practice during COVID-19 pandemic: Position statement from the Indian Endodontic Society, Indian Dental Association, and International Federation of Endodontic Associations. Endod.

[18] Nogueira B.M.L., Silva L.G., Mesquita C.R.M., Menezes S.A.F., Menezes T.O.A., Faria A.G.M., Porpino M.T.M. (2018). Is the Use of Dexamethasone Effective in Controlling Pain Associated with Symptomatic Irreversible Pulpitis? A Systematic Review. J. Endod..

[19] Abu Esba L.C., Alqahtani R.A., Thomas A., Shamas N., Alswaidan L., Mardawi G. (2021). Ibuprofen and NSAID Use in COVID-19 Infected Patients Is Not Associated with Worse Outcomes: A Prospective Cohort Study. Infect. Dis. Ther..

[20] Day C.J., Sandy J.R., Ireland A.J. (2006). Aerosols and splatter in dentistry—A neglected menace. Dent. Update.

[21] Villani F.A., Aiuto R., Paglia L., Re D. (2020). COVID-19 and Dentistry: Prevention in Dental Practice, a Literature Review. Int. J. Environ. Res. Public Health.

[22] World Health Organization (2014). Infection Prevention and Control of Epidemic- and Pandemic-Prone Acute Respiratory Infections in Health Care. https://apps.who.int/iris/bitstream/handle/10665/112656/9789241507134_eng.pdf;jsessionid=C8857696E8E052600F0BEC469D387C20?sequence=1.

[23] Zhang X., Chen X., Chen L., Deng C., Zou X., Liu W., Yu H., Chen B., Sun X. (2020). The evidence of SARS-CoV-2 infection on ocular surface. Ocul. Surf..

[24] Chen C., Zhao B., Cui W., Dong L., An N., Ouyang X. (2010). The effectiveness of an air cleaner in controlling droplet/aerosol particle dispersion emitted from a patient’s mouth in the indoor environment of dental clinics. J. R. Soc. Interface.

